# Antimicrobial Activities of Starch-Based Biopolymers and Biocomposites Incorporated with Plant Essential Oils: A Review

**DOI:** 10.3390/polym12102403

**Published:** 2020-10-19

**Authors:** R. Syafiq, S. M. Sapuan, M. Y. M. Zuhri, R. A. Ilyas, A. Nazrin, S. F. K. Sherwani, A. Khalina

**Affiliations:** 1 Laboratory of Biocomposite Technology, Institute of Tropical Forestry and Forest Products (INTROP), Universiti Putra Malaysia, UPM Serdang, Selangor 43400, Malaysia; mosyafiqrazali@gmail.com (R.S); ahmadilyasrushdan@yahoo.com (R.A.I.); nazrinnurariefmardi@gmail.com (A.N.); khalina@upm.edu.my (A.K.); 2 Advanced Engineering Materials and Composites Research Centre (AEMC), Department of Mechanical and Manufacturing Engineering, Universiti Putra Malaysia, UPM Serdang, Selangor 43400, Malaysia; zuhri@upm.edu.my (M.Y.M.Z.); faisalsherwani786@gmail.com (S.F.K.S.)

**Keywords:** essential oils, starch, biocomposites, anti-microbacterial, biodegradable films, food-packaging applications

## Abstract

Recently, many scientists and polymer engineers have been working on eco-friendly materials for starch-based food packaging purposes, which are based on biopolymers, due to the health and environmental issues caused by the non-biodegradable food packaging. However, to maintain food freshness and quality, it is necessary to choose the correct materials and packaging technologies. On the other hand, the starch-based film’s biggest flaws are high permeability to water vapor transfer and the ease of spoilage by bacteria and fungi. One of the several possibilities that are being extensively studied is the incorporation of essential oils (EOs) into the packaging material. The EOs used in food packaging films actively prevent inhibition of bacteria and fungi and have a positive effect on food storage. This work intended to present their mechanical and barrier properties, as well as the antimicrobial activity of anti-microbacterial agent reinforced starch composites for extending product shelf life. A better inhibition of zone of antimicrobial activity was observed with higher content of essential oil. Besides that, the mechanical properties of starch-based polymer was slightly decreased for tensile strength as the increasing of essential oil while elongation at break was increased. The increasing of essential oil would cause the reduction of the cohesion forces of polymer chain, creating heterogeneous matrix and subsequently lowering the tensile strength and increasing the elongation (E%) of the films. The present review demonstrated that the use of essential oil represents an interesting alternative for the production of active packaging and for the development of eco-friendly technologies.

## 1. Introduction

**Figure 1:**
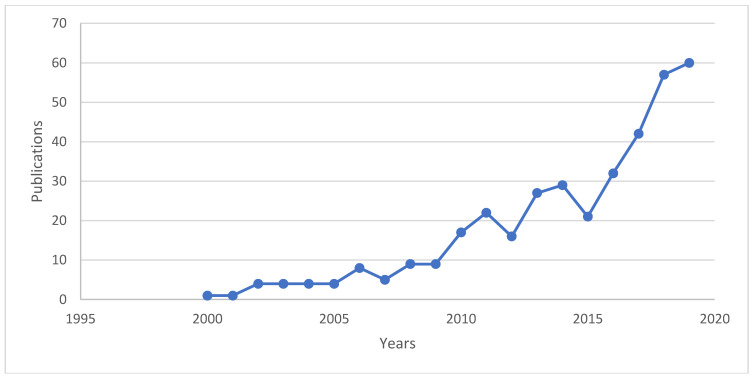
The trend research on starch based composite reinforced essential oil as biopolymer (SCOPUS, August 2020, Keywords: starch, essential oil).

Food packaging can prevent food damage [1,2,3,4,5,6]. It is essential to select the proper materials and packaging techniques to retain food quality and freshness [7,8,9]. Edible film packaging is new and environmentally friendly food preservation technique compared to conventional packaging. The biodegradable film retains food quality and is environmentally friendly [7,8,9,10,11,12,13]. Although several kinds of new polymers such as (polylactic acid (PLA), polyhydroxyalkanoates (PHA), polycaprolactone (PCL), and poly(ethylene adipate) (PEA) are industrially produced, agricultural based polymers are the most investigated by researchers, mainly polysaccharides [14,15,16,17,18,19,20,21,22,23,24]. Numerous researchers reported that reinforcement of essential oil into starch based packaging can increase their stability and retain their flavour and functional properties [25,26,27,28,29,30,31,32,33]. Figure 1 shows the trend of study on the starch biopolymer incorporated essential oils. It is possible to add essential oils (EOs) as additional materials to enhance the antimicrobial properties of the edible film. An edible film with antimicrobial characteristics has potential to prevent contamination in food product [34]. The usage of small amounts of EOs can reduce cost while having higher mechanical properties to allow the film to maintain its structure for a long time. The incorporation of antimicrobial agents into starch based films provide benefits, such as low antimicrobial concentrations and low diffusion speed. Antimicrobial activity was observed to be more active against the bacteria tested than the control film to extend product shelf life [35]. Antimicrobial packaging is among the best practices aimed at increasing food shelf life by inhibiting microbial growth. EO components (EOCs) extracted from aromatic plants have a broad spectrum of antimicrobials (bacteria, fungi, and yeast) and have proven their efficiency as antimicrobial additives in food packaging materials [36,37,38]. One of the drawbacks of using EOCs in food packaging is their strong odour that can change fresh3 food’s organoleptic properties. The techniques used in the recycling program were unable to cater for excessive amount of non-degradable waste accumulated due to different challenges and the funds required during recycling [22,28].

Starch-based polymers have real potential as feedstock to manufacture bio-plastic film on a large scale [39]. Starch-based edible film has similar mechanical properties and the transparent appearance with conventional plastic [40,41,42,43]. On the other hand, these polymers have poor physical properties, such as fragile structure, short-term stability, low mechanical strength, high gas permeability, low heat distortion temperature, low water barrier resistance, and low melting viscosity for subsequent processes [31]. Oliveira et al. [44] worked on the development and characterization of biopolymer films based on bocaiuva flour reinforced clove essential oil. The addition of the essential oil increased the elongation at break, indicating it acted as plasticizer, which has higher value of water vapor permeability (WVP) [44]. Thus, this review paper highlighted the antimicrobial effect of starch-based biopolymer incorporated with essential oil. Besides that, mechanical, thermal and barrier properties of the biopolymer were reviewed.

## 2. Starch-Based Polymer Film Incorporated with Antimicrobial Plant Essential Oils

### 2.1. Antimicrobial Properties of Starch-Based Polymer Film Incorporated with Antimicrobial Plant Essential Oils

As a current antibacterial agent, EO has a long history of use for antibacterial activity [45,46]. Table 1 shows the starch-based polymer film incorporated with antimicrobial plant EOs for food packaging applications. Currently, EO has increased its reputation as a harmless, natural, and active antibacterial. Many sectors such as pharmaceutical and cosmetic products have proposed to use it externally as the primary antimicrobial or as a natural preservative [47,48,49,50,51,52,53,54,55]. The tests of *Escherichia coli* cells or other microbes using an electron microscopy after having contact with EOs discovered a damage of cellular electron-dense material and coagulation of cytoplasmic parts. [45,56]. In addition, various types of antimicrobial EO additives and concentrates have been applied to the composite film to overcome microbial activity in the film. Improving these completely biodegradable packaging films is helpful in resolving the ongoing environmental issues and gradually replacing traditional packaging materials that are commonly used [57,58].

The best concentrations of EOs that are effective against different microorganisms depend on the type’s of EOs and biopolymers used as shown in Table 2. According to the study conducted by Helal et al. [59], the results showed that the EOs from *Origanum vulgare* showed the lowest minimum inhibitory concentration (MIC) of 0.025 mg/mL for *Staphylococcus aureus* and *Streptococcus pyogenes* if compared with *Mentha cervina* (0.05 mg/mL) and *Ocimum basilicum* (1.6 mg/mL). Besides that, the EOs from *O. vulgare*, *Ocimum basilicum* and *Mentha cervina* were the most active against all isolates, with the inhibitory zone ranges between 17 and 45 mm. Meanwhile, *Mentha pulegium* L. demonstrated moderate activity from 13 to 45 mm. This might be attributed to the presence of bioactive metabolites of various chemical types, such as 1-Terpineol (19.68%), L-Linalool (60.97%), and Pulegone (58.54%) of *O. vulgare*, O. basilicum and *M. cervina*, respectively.

There are large number of published studies describing the role of EOs as antimicrobial agents as shown in Table 2. Ferreira et al., [60] conducted a study on the evaluation of in vitro antimicrobial activity of six types of EOs against *S. aureus*, *E. coli*, and *S. enterica*. Among the six EOs studied, three EOs (sweet orange, rosemary, and cinnamon) did not show any significant differences between Gram-positive and Gram-negative bacteria, thus revealing a good spectrum of action (Table 1) [60]. The authors summarized that the Gram-negative bacteria (SE, EC) were less sensitive to the action of EOs when compared with Gram-positive bacteria (SA). This phenomenon might be attributed to the difficulty of the compounds to act on the complex structure of the cell wall of Gram-negative bacteria.

Song et al. [56] investigated the antimicrobial properties of biodegradable films process through injection of EOs into the starch films of different concentrations of lemon EOs (LO) and surfactants (Span 80, Tween 80). Active packaging corn/wheat (CW) starch-based biopolymer can be made by reinforcing it with lemon oil and surfactant. The result showed that LO and surfactant would affect the structure and antibacterial properties of biopolymer film. Besides that, emulsification was observed in CW-LO-T/S films, where the Tween 80 and Span 80 showed different effects on the films’ colour, solubility, and water vapor permeability. The most surprising aspect of the data was that, higher LO contents had larger inhibition zone and microporous holes.

**Figure 2:**
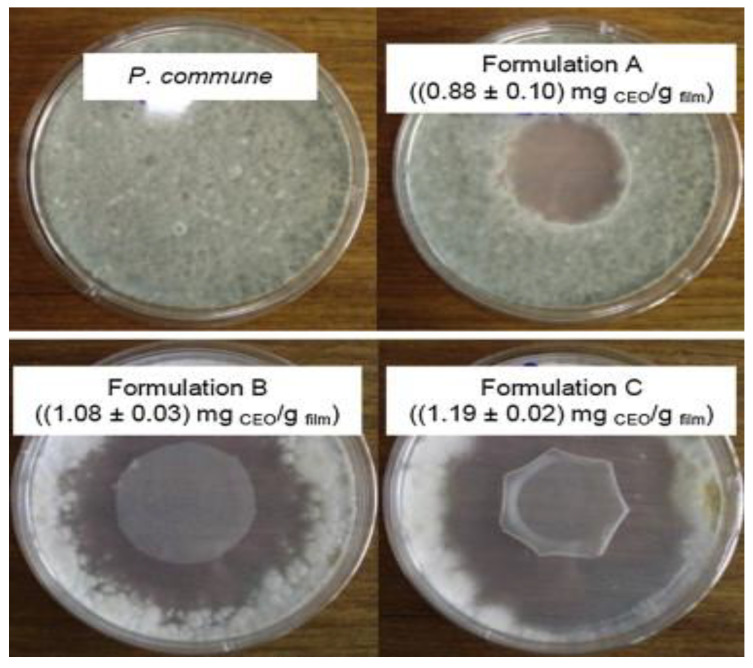
Petri dishes with circular disks of film incorporated with three different concentrations of cinnamon EOs, showing the inhibitory zone against *Penicillium commune* in comparison with the Petri dish without the active film. (Reproduced with a copyright permission from Souza et al. [7]).

Souza et al. [7] studied the impact of EOs of clove and cinnamon combined with cassava starch films to develop an active packaging. The result exhibited that all the films, containing different amounts of EOs, showed effective antimicrobial activity against fungi, *Eurotium amstelodami* and *Penicillium commune* in bread products. The minimum amount of oil used to inhibit 100% of the microorganisms were 2.0 g/100 g and 16.0 g/100 g for cinnamon and clove EOs, respectively. Figure 2 shows the inhibition of *P. commune* caused by active films produced by three different concentrations of cinnamon Eos, which are 0.88 mg/g, 1.08 mg/g, 1.19 mg/g. From Figure 2, it can be seen that no inhibition zone against the microorganisms was observed for the control film, as compared to the film disks incorporated with cinnamon EOs. A better inhibition was observed with higher content of cinnamon essential oil. Besides that, according to Souza et al. [7] in their research, they had concluded that the *E. amstelodami* was more sensitive to cinnamon EOs because its inhibition were greater, reaching approximately 91% of inhibition with the highest concentration used, compared to other microorganisms.

No microorganism inhibition zone for film disk without the inclusion of essential oil (control film) was found. *E. Amstelodami* became more susceptible to cinnamon EOs because its inhibition was stronger, achieving about 91% of the maximum concentration inhibition reported. Figure 2 shows the inhibition of *P. commune* from active films produced with three separate contents of cinnamon EOs [7].

Iamareerat et al. [57] studied the biodegradable starch-based films combined with cinnamon EOs and sodium bentonite clay nanoparticles based on their anti-bacterial potential to evaluate the life span of meatballs. The control films, exceeded the limit of 48 h as compared to meatballs, packed in cassava starch film integrated with cinnamon oil and nanoclay at ambient temperature that substantially inhibited microbial growth in foods up to 96 h below the Food and Drug Administration (FDA) limit (10^6^ CFU/g). The highest acceptable amount of total plate count in meat is 10^6^ CFU/g, as per the (FDA) report. Besides that, it is reported by Iamareerat et al. [57] that the meatballs packed in cassava starch film with cinnamon oil and sodium bentonite stayed under the highest permissible level until 96 h, whereas the control film exceeded the maximum permissible level within 48 h [36].

Silveira et al. [61] studied the development of active cassava starch incorporated with natural antimicrobial tea tree essential oil based on the physical, structural, and antimicrobial properties of the film. The antimicrobial activity of the tea tree oil at concentration of 0.08% was 77% to inhibit S. aureus while 65% to inhibit C. albicans. The result showed biological growth inhibition needed higher oil concentration or the isolated action of tea tree oil [61]. Tea tree oil contains terpinen-4-ol and 1,8-cineole as the evidence to inhibit *S. aureus* [61].

Utami et al. [62] reported a research on characteristics of edible film and quality of fresh beef for the effects of cinnamon bark essential oil. It was reported that the higher the concentration of essential oil, the larger area of inhibition zone. Cinnamon essential oil contains about 85% cinnamaldehyde, it can react with enzymes and proteins as well as the membrane phospholipid bilayer by penetrating the membrane of microorganisms, which causes disruption of microbial and enzyme systems or interferes with the function of genetic materials [62].

### 2.2. The Mechanisms of Action of EOs in Antimicrobial Activity

Several researchers have conducted comprehensive studies on the EOs antimicrobial properties as well as their compounds. Although the mechanism of action of EOs components has been well explained in numerous works in the past, extensive knowledge of most of the compounds and their mechanical properties is still lacking [63]. This knowledge is very important to determine the effect of EOs on different microorganisms, their mechanism in combination with other antimicrobial EOs, as well as their interaction with food matrix components. Chouhan et al. [64] performed a comprehensive review on the antimicrobial activity of selected EOs. They discussed different antimicrobial agents from different sources of EOs for microbial resistance purpose. They also discussed the mechanisms of action of different Eos, for instance, cinnamon. It targeted microorganisms such as *E. coli* and *S. aureus* and the mechanism of action was the disruption of cell membrane. Other possible mechanisms of actions included permeabilized membrane and cell wall damage [65].

Moreover, Saad et al. [66] reported that the antimicrobial activities of EOs such as antibacterial, antifungal and antiviral activities were extensively studied in the past but admitted that very limited works have been carried out on their mechanisms of action. Once the mechanism of action is determined, it helps in the selection of the ideal conditions of EOs during growth, harvest, and oil extraction [66]. The work of Li et al. [67] was concerned with developing EOs from the finger citron EOs (FCEOs), *Citrus medica L. var. sarcodactylis*. It was used as an antimicrobial against food-borne bacteria. Li et al. [67] further reported that the mechanisms of action were studied by the morphological change in bacteria using important techniques, such as scanning electron microscope (SEM), and time-kill analysis. The bacterial morphology tested had changed and was seriously damaged by the concentration and exposure time of FCEO. FCEO exhibited a significant reduction in bacterial growth rate and led to cell wall lysis, intracellular material leakage, and consequently, cell death.

Tao et al. [68] studied the effects of EOs from the leaves of Phyllostachys heterocycla cv. pubescens (bamboo) against food-borne pathogens, such as Gram-negative (*E. coli*), Gram-positive (*Bacillus subtilis* and *Staphylococcus aureus*), yeast (*Saccharomyces cerevisiae*), and bacteria. Different characterization of the EOs were performed. The mechanism of action of bamboo leaf EOs was described as a disruption to the pathogen’s membrane integrity.

To assess the resistance to microbial degradation of plastic materials, some of the standard methods [69] had been formed, such as:

1. IEC 68-2-10: Clear techniques for monitoring the environment
2. EN ISO 846: Plastics-appraisal of microorganisms’ action
3. ASTM G21-90: Standard practice for determining synthetic polymeric fabric’s resistance to fungi
4. ASTM G22-76: Standard practice for assessing immunity to bacteria in synthetic polymeric products.

There is a variety of test methods that can be applied. There is no standard method for determining antimicrobial polymer efficacy [70]. The method for inorganic antimicrobials, such as silver replaced zeolites was established. The goal was to define the plastic’s resistance to microbial growth, but it also helped to find if polymers were self-sterilizing.

Interest in natural antimicrobials is also driven by the fact that the requirements of international regulatory agencies for toxicological evaluation of novel direct food antimicrobials are generally very strict. Toxicological testing for new synthetic substances could take several years and millions of dollars to be explored by researchers around the world. A payback may be necessary for some kind of food additives like artificial sweeteners. It is less likely that it would be feasible to gain clearance for meat antimicrobials. An argument often used to justify natural antimicrobials is that they will produce “green” labels, like one in the list of ingredients with few or no “synthetic” additives. Although this reasoning might be valid, it should originate from natural sources, such as the following [71]:

1. Acetic acid from vinegar
2. Benzoic acid from cranberries, plums, prunes, cinnamon, cloves, and most berries
3. Lactic acid from lactic acid bacteria
4. Propionic acid from Swiss cheese (*Propionibacterium freudenreichii ssp. Shermanii*)
5. Sorbic acid from rowanberries

If an effective antimicrobial has been found from natural sources, it is more cost-effective than synthesizing it in the laboratory. This rationale also leads consumers to believe that food additives currently in use are highly toxic and should be averted [71]. 

### 2.3. Mechanical Properties of Anti-Microbacterial Agent Incorporated Starch Biopolymer 

Various studies have assessed the efficacy of EOs with starch-based biopolymer, as shown in Table 3. The mechanical properties of starch reinforced by the anti-microbacterial agent were tested on tensile strength, Young’s modulus, and elongation at break. From Table 3, it can be observed that potato starch mix of 2% lavender EOs had the highest tensile strength for this study. Cassava starch added with cinnamon EOs resulted in the highest value of elongation at break. According to Table 3, the tensile strength recorded at 2% of lavender EOs was 62.8 ± 1.9 MPa, while elongation at break was 19.5% ± 2.6%.

To develop an effective packaging, Souza et al. [7] conducted a research on the impact of cinnamon and clove essential oils, combined with cassava starch films. The tensile strength (TS) and elongation at break (E) ranged from 2.32 ± 0.40 to 1.05 ± 0.16 MPa and from 264.03% ± 35.06% to 191.27% ± 22.62%, respectively. They were compared to the control film which presented higher TS of 3.96 ± 0.60 MPa and lower E of 123.61% ± 19.57%. Song et al. [56] had conducted a research on the molecular structure and mechanical properties (tensile strength (TS) and elongation at break (E)) of active packaging corn/wheat (CW) starch-based biopolymer reinforced with lemon oil and surfactant. The mechanical properties (tensile strength and elongation at break) of corn/wheat reinforced lemon EOs with Tween 80 and Span 80 were 11.16 ± 1.03 MPa and 11.50 ± 1.0 MPa, and 626.50% ± 0.39% and 32.20% ± 0.55%, respectively. In another study conducted by Iamareerat et al. [57], a biodegradable cassava starch-based film was incorporated with cinnamon EOs and sodium bentonite clay nanoparticles based on mechanical properties to assess the shelf life of meatballs. From Table 3, the film with 1.5% of EOs had high value of tensile strength, which was 0.63 ± 0.09 MPa, as compared to other percentage of EOs.

A considerable amount of literature has been published on starch biopolymer reinforced with EOs. Jamróz et al. [58] conducted a research on the addition of lavender EOs (LEOs) to starch, furcellar and gelatin (S/F/G) films with respect to mechanical properties. The tensile strength reduced significantly with increasing oil concentration, while the elongation at break parameters remained. Tensile strength decrease might result in lower mechanical strength.

Another study had been conducted by Cano et al. [73], in which they investigated the physical and antimicrobial properties of starch-PVA blend film. Influenced by the introduction of natural antimicrobial agents, the films were worthy and innovative alternatives in food packaging applications. The films’ mechanical performances were also changed by the incorporation of oils, but this was released at the maximum oil ratios. At the minimum oil concentration, the mechanical properties of the films were in the range of those that were commercially available. Based on Table 3, the tensile strength of the film for oregano EOs and neem EOs were 26 ± 2 MPa and 21.5 ± 1.0 MPa, respectively. This showed that the film containing oregano EOs had higher value of tensile strength than the film that contained neem EOs. The better film that represented a novel and good alternative for use in food packaging was the film that contained oregano EOs [1].

**Figure 3:**
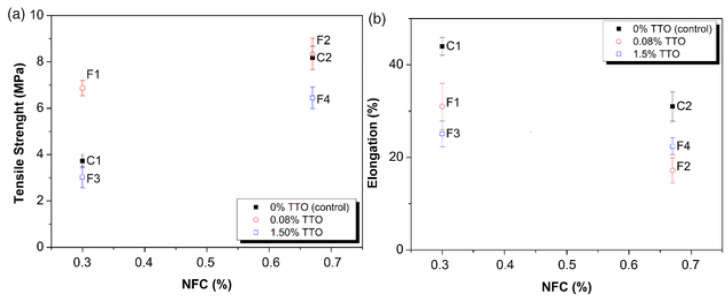
TS (**a**) and Elongation (**b**) of the films for different levels of NFC and TTO. Control films: C1 (0.3% *m/v*, NFC); C2 (0.67% *m/v*, NFC); and films with TTO: F1 (0.3% *m/v*, NFC, 0.08% *v/v*, TTO); F2 (0.67% *m/v*, NFC; 0.08% *v/v*, TTO); F3 (0.3% *m/v*, NFC; 1.5% *v/v*, TTO); F4 (0.67% *m/v*, NFC; 1.5% *v/v*, TTO). (Reproduced with a copyright permission from Silveira et al. [61]).

Besides that, Silveira et al. [61] reported the mechanical properties of the film at 1.5% of the tea tree oil (F3) was 3.03 ± 0.46 MPa for the tensile strength, while 25.1% ± 2.8% for elongation at break. From the Figure 3 it can be observed that the addition of 0.08% TTO (F1) increased the tensile strength (TS) while formulations with 1.5% TTO (F3) decrease the TS comparing to control films, for both levels of cellulose nanofiber (NFC). Besides that, the incorporation of 0.08% TTO in a 0.3% NFC-starch matrix caused a significant increase of 184% in the TS, raising from 3.37 to 6.87 MPa, for films C1 and F1, respectively. The present of NFC can increased the mechanical properties of the films due to its intrinsic properties as reported by numerous authors [84,85,86,87,88,89,90,91,92,93]. In addition, the result was slightly decreased for tensile strength as the increasing of essential oil while elongation at break was increased. This result shows that low concentrations of TTO can improve the mechanical properties of film. A strong interaction between the oil and the NFC–starch matrix can produce chemical modifications, for example, crosslinking effect. Whereas, the increasing of essential oil caused the reduction of the cohesion forces of polymer chain, creating heterogeneous matrix and subsequently lowering the TS and increasing the elongation (E%) of the films [61].

Mechanical properties of the tapioca film incorporated with cinnamon bark essential oil were researched by Utami et al. [62]. The tensile strength was increased only up to 1% concentration of essential oil, after that the tensile strength started to decrease by the increasing of the concentration of essential oil. Changes in the water content of the film caused the tensile strength to improve. The increasing concentration of the essential oil decreased the tensile strength of the films. Addition of essential oil to the film solution weakened the film tensile strength, which induced the development of a heterogeneous film structure [62]. The elongation at break also decreased by the increasing of the essential oil concentration. Essential oil created a compact structure by improving continuity in a network of polysaccharides, which caused the elongation at break to decrease. Gao et al. [94] reported the mechanical properties on corn/octenylsuccinated starch incorporated with soybean oil. The tensile strength of the film incorporated with 0.5% soybean oil was 5.21 MPa, while elongation at break was found to be at 36.54%. The strong interaction between the starch molecules produced a cross-linker effect, which decreased the molecular mobility of the starch [94].

### 2.4. Water Barrier Properties of Anti-Microbacterial Agent Reinforced Starch Biopolymer

Water barrier is an important properties for food packaging application [98,99,100]. Cano et al. [73] carried out the experiment by mixing pea starch with PVA (polyvinyl alcohol) reinforced with oregano EOs (OEOs) and neem EOs (NEOs). The moisture contents of the S-PVA blend films were 4.40% and 4.33% for 1% and 10% of the solution, respectively. Films containing bioactive substances showed lower levels of moisture after one week of processing. Films with the highest amount of neem oil hit the balance after one week of processing. Based on the concentration, the different behaviours of the films contributed to the specific relationship between the matrix and the oil components. Through hydrogen bonding, oil components could be linked to hydroxyl groups accessible in starch and PVA, thus limiting the interactions between polymer and water, and resulted in a decrease in film-moisturizing speed [101]. The integration of oils into the film did not lead to significant variations in the water vapor permeability (WVP) standards. Consequently, the change of structure caused by oils in the polymer matrices did not imply the significant variations in their barrier potential for water vapor. Such active films containing oregano EOs thus provide a novel and effective alternative for use in food packaging. Such films could be used to prolong the life of bread and cheese, and, by using only natural compounds with antimicrobial activity, as a coating agent for bananas and mangos. Oil incorporation cannot affect the capacity of water sorption and water vapor barrier properties of S-PVA films, particularly at the highest concentrations, but can decrease their transparency and gloss [73].

Besides, Resianingrum et al. [1] proved that the introduction of lemongrass EOs had no significant effect on the cassava starch-based edible film’s water vapor transmission rate. The main factor was the edible film’s high water vapor transmission rate because the hydrophilic component was higher than the hydrophobic one [1]. According to Djunaedy et al. [102], the water vapor permeability of edible film decreased if the edible film hydrophobic component was increased. The hydrophobic properties will increase, and the hydrophilic properties will decrease with the introduction of hydrophobic compounds to an edible film solution. Thus, it is assumed that the hydrophobic properties of the cassava starch-based edible film with the addition of lemongrass EOs did not change significantly, and the addition of lemongrass EOs did not significantly vary the rate of water vapor transmission on the film. The hydrophilic ratio of the edible film was still higher than the hydrophobic ratio [102].

The moisture content of the various films, which were a mixture of maize starch with lemon essential oil (LEOs) was analysed by Song et al. [56]. The water content in the films decreased with increasing LEOs concentrations. This may be due to the interaction between the two functional groups of LEOs and the starch, which decreased the interaction between polysaccharides and water, resulting in a reduction in water content. The inclusion of surfactants in the polymer films affected their water content. The water content decreased significantly when Span 80 was applied to the films (P < 0.05). Hydrophobic surfactants such as Span may reduce water vapor adsorption of hydroxypropyl methylcellulose films and result in decreased water content. In this study, the low concentration of Tween in the film formulation can cause this phenomenon. The most important point in their suitability for food packaging is the films’ WVP [56]. Other researchers noted an increase in the WVP of films containing EOs [101]. We can also see that by adding surfactants in the film matrix, the films’ WVP values were decreased. The results revealed that the surfactants had a major impact on the variation in WVP values. The WVP values decreased with the inclusion of starch/decolourised hsian-tsao leaf gum composite films. The film containing only Tween 80 displayed a higher water content and WVP as compared to the film containing only Span 80. The incorporation of LEOs resulted in a reduction in water content and WVP [56].

Regarding the characteristics of the barrier properties, Souza et al. [7] tried to increase the amount of glycerol, emulsifier, and EOs for cinnamon effect, thus resulting in higher values of both permeabilities. Due to this finding, an increase in glycerol content in the starch films produced in this study could also lead to a decrease in the properties of the barriers studied. A lepidic component added to the formulation can act as a barrier in the films. Therefore, the responsible agent for elevating permeability values was not supposed to be cinnamon EOs. Water vapor permeability (WVP) and oxygen permeability coefficient (P’O_2_) of films combined with cinnamon EOs ranged from (9.78 ± 1.40 to 14.79 ± 2.76) g mm m^−2^ d^−1^ kPa^−1^ and from (27.50 ± 0.60 to 143.47 ± 8.30) × 10^9^ cm^3^ m^−1^ d^−1^ Pa^−1^, respectively. The emulsifier was possibly responsible for the WVP based on previous observations. It should be noted, however, that its presence in the film phase is important, as it facilitates the incorporation of the antimicrobial agent in the aqueous solution, resulting in a homogeneous polymer matrix [7].

The water content of the potato starch film was obtained by Jamróz et al. [58] at the concentrations of 0%, 2%, 4%, and 6% LEOs. There was a reduction in the absorption of water in the film when LEOs were added as compared to the control test. The film’s water content with the addition was 68.81–72.28%. The film with the highest oil concentration (6%) had an order of 68.81% for water absorption. The control film’s water content was 74.48%. The reduction in water absorption films with a concentration of 2%, 4% and 6% LEOs, which were different from the control sample, was 2.95%, 5.8%, and 7.61%, respectively as shown in Table 3. This was due to the interactions between LEOs, potato starch, furcellaran, and gelatin functional groups that restricted polysaccharide-water-protein interactions, leading to a reduction in water content.

Silveira et al. [61] reported the trend of WVP of the film found to be decreasing by the increasing of the tea tree essential oil. The decreasing of WVP indicated the cohesion of polymeric chain formed. The strong intermolecular interactions that created barriers to water vapor diffusion through the matrix. Furthermore, the presence of the oil droplets caused it to become hygroscopic nature [61]. Water vapor permeability (WVP) of the tapioca film incorporated with cinnamon bark essential oil reported by Utami et al. [62] was decreased by the increasing concentration of essential oil. The better protection from moisture of food has the criteria of lower value of WVP [62]. Besides that, water vapor permeability occurs only in the hydrophilic condition. According to Gao et al. [94] the water vapor permeability (WVP) for the corn/octenylsuccinated composite film incorporated with soybean oil slightly decreased, according to the increasing of the concentration of essential oil. The hydrophobic property of the film by the decreasing of the WVP value weakened the ability of film to bind with water [94]. The incorporation of soybean oil into the film, formed a discontinuity in the hydrophilic phase that caused an increasing of the effective path length for diffusion and decreased the film’s WVP [94].

### 2.5. Antimicrobial Activities of Nanocellulose Reinforced Starch Bionanocomposites with EOs

Costa et al. [103] studied the characterization and determination of the bifunctional efficacy of active packaging films produced with starch and glycerol reinforced with cellulose nanocrystals (CNC). This film was activated by extracted red propolis (ERP). The use of the active films for packaging cheese curds and butter were evaluated on the antimicrobial and antioxidant efficacy, respectively. The addition of the cellulose nanocrystals will increase the mechanical strength of the films and can reduce the water permeability and water activity. The testing was done on their total phenolic compounds and mechanical properties. Besides, the films were characterized by using moisture, water-activity analyses, and water vapor permeability tests.

**Figure 4:**
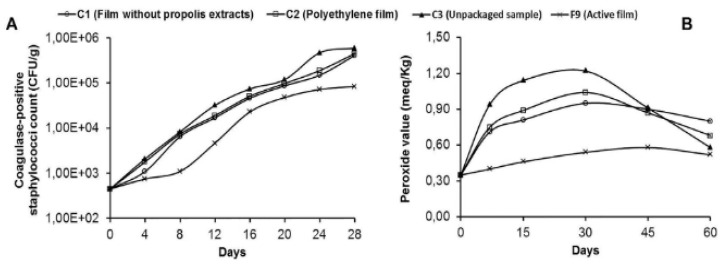
Antimicrobial efficacy (**A**) and antioxidant efficacy (**B**) of the active film during storage. [103] (Reproduced with a copyright permission from Creative Commons Attribution License).

The number of colony-forming unit (CFU) specified that the ERP in the active film had an inhibitory activity against the microorganism, as shown in Figure 4A. It was assessed that the lowest number of CFU was reached during the storage period. The effectiveness of the compounds can be reduced by the interaction between the polymer groups and active compound of the embedded agent. It can reduce or even prevent the diffusion of active compounds to the system [103]. The result from Figure 4B suggested that these films did not provide any protection to the product during storage. The mechanical reinforcement achieved by the addition of nanocellulose to the films reduced both the films’ permeability to water vapor and their permeability to gases. It would have a direct effect on the oxidation of the stored product. The use of antioxidants in packaging was the main reason for the food lipids to delay a significant accumulation of free radicals and thus improve oxidative stability [103].

**Figure 5:**
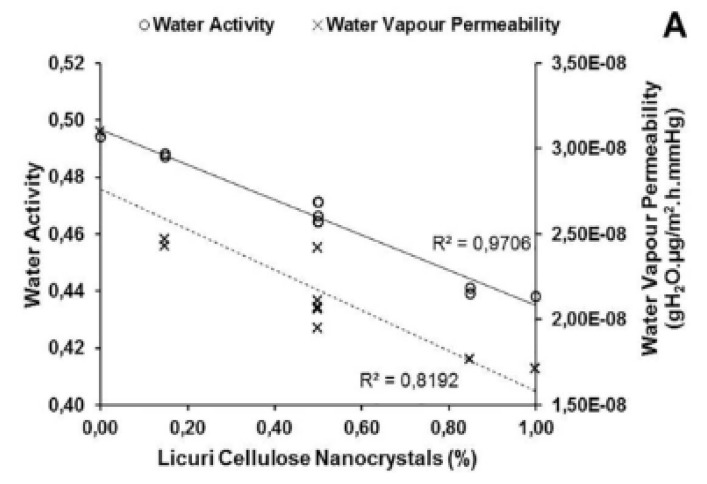

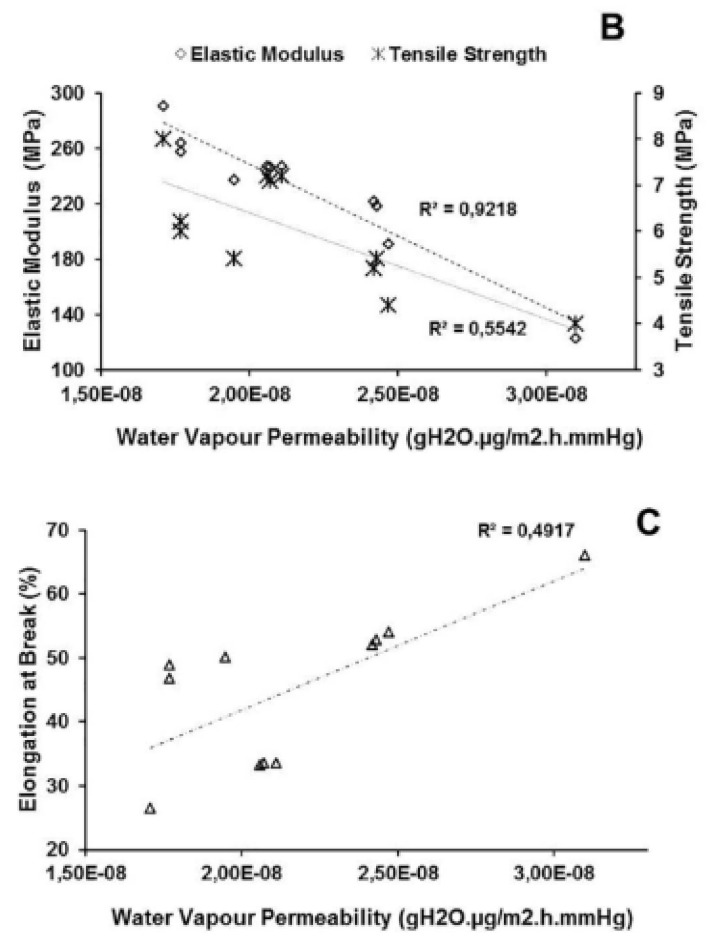
(**A**) Linear correlation between the CNC and the physico-chemical properties, (**B**) water vapor permeability (WVP), and, (**C**) mechanical properties of the films [103]. (Reproduced with a copyright permission from Creative Commons Attribution License).

CNC decreased the water availability within the matrix of the nanocomposite material and had a relevant role in controlling water availability due to the rise in the number of hydrogen bonds with the polymeric matrix and plasticiser [104]. The WVP values and the CNC concentrations of the films showed an inverse proportional and linear relationship (Figure 5A). At any concentration, the cellulose nanocrystals in the films were intended to reduce the WVP by providing a physical barrier, reducing the free spaces in the polymer matrix, restricting the passage of vapors and inhibiting water absorption [103]. The degree of crystallinity of the cellulose nanoparticles and the strong hydrogen bonds formed within the matrix restricted access, and hence, the permeability of water through the films [105]. Figure 5B,C show the linear correlation between the WVP values and mechanical properties of the films. There was a linear and inverse proportional correlation between the WVP and elastic modulus of the films [103]. The increased permeability of the films significantly reduced the films’ stiffness and their ability to stretch. This correlation mainly indicated that the addition of CNC can linearly reduce the WVP and increase the elastic modulus of the films, thus making the films more reasonable with standard products.

## 3. Antimicrobial Properties of Polymer Composites Reinforced Essential Oil

Polymers are widely used in many applications, such as in food packaging. Commercial polymers used fossil fuels as the main source, causing the increasing environmental concern regarding their use. To replace the petroleum-based polymers, there are many researches on the usage of bio-based materials. There are several types of renewable sources for biopolymers, including polysaccharide such as pectin, cellulose, starch, gelatin, and alginate [89,106,107,108,109,110].

Recently, many researchers have done the researches on the utilization of chitosan as biopolymer. Although chitosan is a biopolymer for food packaging, it has no significant antimicrobial and ambiguous antioxidant activities. Improvement needed as the antimicrobial activity of chitosan could expand its applications in food packaging. Essential oils (EO)s are used in different industries that are produced by angiosperm plants. Only aromatic plants will be used as sources of EOs. EOs are secondary metabolites which could be derived from different parts on the plant, including flowers, buds, leaves, fruits, twigs, bark, seeds, wood, rhizome, and roots. Nowadays, EOs extracted from plants as active agents have received much attention to be incorporated into biodegradable active films. Various researches have shown that the incorporation of EOs into biopolymers resulted in the increasing of the film’s antimicrobial activity and antioxidant properties. Besides that, the water vapor permeability was decreased. Table 4 summarizes the essential oils reinforced various polymer composites that have been successfully used in food packaging for improved efficiency.

## 4. Conclusions

Antimicrobial biodegradable polymeric films can substitute the conventional packaging material to reduce environmental hazards and extend the shelf life of food products. Due to their environmentally friendly and anti-microbial properties, EO-starch-based films has the potential to be used in food packaging product applications. Although starch films are most likely to be applied as alternative packaging materials, they must possess adequate mechanical properties to resist maximum external stress and preserve the integrity and barrier properties, like food packaging application. According to antimicrobial activity results, almost all the essential oil (i.e., lemon, peppermint, cinnamon, lavender, mexican oregano, oregano, neem, lemon grass, tea tree, *Lavandula angustifolia, Mentha pulegium*, turmeric, lime, *Origanum vulgare L.*, *Ziziphora clinopodioides*, grape seed, and *Zataria multiflora Boiss* etc.) can be used as antimicrobial agent against the microorganism and fungi selected. It can be observed that the incorporation of lemon, lemon grass and tea tree essential oil within starch-based films are very effective against *E. coli*, *S. aureus* and *A. niger*, respectively. Whereas cinnamon essential oil is very effective against *C. albicans, P. commune*, and *E. amstelodami* microorganisms. It can be concluded that a better inhibition was observed with higher content of essential oil. Even at minimum concentration applied into the film formulation, essential oil showed inhibition against microorganisms, which was considered an important result since that higher concentrations could imply a sensorial impact, altering the natural taste of the food packaged by exceeding the acceptable flavor thresholds. Besides that, the incorporation of essential oil within starch-based film would affect the mechanical properties of the films. It can be summarized that the high concentration of the essential oil within starch-based polymer can reduce the mechanical properties of the film. The increasing of essential oil caused the reduction of the cohesion forces of polymer chain, creating heterogeneous matrix and subsequently lowering the tensile strength and increasing the elongation at break value of the film. The development of such fully biodegradable packaging films will help to tackle the current environmental problems caused by the disposal of non-biodegradable plastics. The successful production of such green materials would provide an opportunity to enhance the world’s farmers’ quality of life by generating non-food economic development sources for rural areas.

## Figures and Tables

**Table 1:** Antimicrobial activity of EOs by disc diffusion assay [60].

EOs	Diameter of Inhibitory Zone (mm)		
	Bacteria		
	*Staphylococcus* *aureus (SA)*	*Escherichia* *coli (EC)*	*Salmonella* *enterica (SE)*
Cinnamon	18.2 ± 1.8	19.7 ± 1.8	15.5 ± 2.8
Pepper mint	12.4 ± 2.3	5.8 ± 0.3	NA
Rosemary	9.6 ± 2.5	7.9 ± 1.8	9.0 ± 1.2
Sweet orange	7.8 ± 0.6	8.7 ± 1.4	7.9 ± 0.7
Tahiti lemon	6.8 ± 0.4	NA	NA
Ginger	6.0 ± 2.2	NA	NA

NA = not applicable.

**Table 2:** Starch-based polymer film incorporated with antimicrobial plant EOs for food packaging application.

Starch	EOs	Inhibition Zone (%)		References
		***S. aureus Gram* (+)**	***E. coli Gram* (-)**	
Corn/Wheat	Lemon	47.72 ± 2.87	45.56 ± 3.15	[56]
Corn/Wheat	Lemon	45.89 ± 3.36	45.34 ± 3.48	[56]
		***L. monocytogenes***		
Modified Starch	Peppermint oil	-	-	[25]
		***P. commune***	***E. amstelodami***	
Cassava	Cinnamon	25.94 ± 5.72	91.06 ± 15.48	[7]
		***Escherichia coli***	***S. aureus***	
Cassava	Cinnamon	12.17 ± 0.29	11.17 ± 0.29	[57]
		***E.coli ATCC* 25922**	***S.aureus ATCC* 25923**	
Potato	Lavender	-	-	[58]
		***Aspergillus niger***	***Penicillium spp.***	
Amaranth/Chitosan/Starch	Mexican Oregano	-	-	[72]
		***A. niger***	***P. expansum***	
Pea	Oregano	-	-	[73]
Pea	Neem	4.35 ± 0.10	4.34 ± 0.06	[73]
		***Pseudomonas fluorescens***	***Aspergillus niger***	
Cassava	Lemon Grass (0.5%)	25.89 ± 0.12	11.97 ± 0.176	[1]
		***S. aureus Gram* (+)**	***Candida albicans***	
Cassava	Tea tree (TTO) (1.5%)	77 ± 0.24	65 ± 0.24	[61]
		***Pseudomonas fluorescens***		
Tapioca	Cinnamon bark (2.0%)	28.6 ± 0.85		[62]
		***S. aureus***	***L. monocytogenes***	
Potato	*Lavandula angustifolia* (0.5%)	13.69 ± 0.89	17.25 ± 0.59	[74]
Potato	*Mentha pulegium* (0.5%)	21.01 ± 0.42	22.90 ± 0.90	[74]
		***A. niger***		
Cassava	Tumeric (0.5%)	17.02 ± 0.42		[75]
		***Escherichia coli***	***Candida albicans***	
Sodium Alginate (NaAlg)	Cinnamon (50%)	6 ± 0.12	13 ± 0.06	[76]
	Lavender (50%)	0.5 ± 0.89	2 0.90	
	Tea tree (50%)	0.5 ± 0.59	1.5 ± 0.10	
	Peppermint (50%)	1 ± 0.06	2 ± 0.12	
		***Escherichia coli***	***S. aureus***	
lime bagasse pectic extract	Lime (0.5%)	11.8 ± 1.0	13.5 ± 0.8	[77]
lime pomace pectic extract	Lime (0.5%)	9.5 ± 0.9	8.3 ± 0.8	
		***Escherichia coli***	***S. aureus***	
Fish gelatin/Chitosan nanoparticle	Origanum vulgare L. (1.2%)	33.00 ± 1.00	26.33 ± 0.57	[78]
		***B. Subtilis***	***S. aureus***	
Chitosan	Ziziphora clinopodioides (1%)	5.4 ± 0.1	6.1 ± 0.1	[79]
	Grape seed (1%)	1.7 ± 0.1	3.1 ± 0.1	
Gelatin	Ziziphora clinopodioides (1%)	4.3 ± 0.2	5.6 ± 0.1	
	Grape seed (1%)	1.3 ± 0.1	2.1 ± 0.1	
		***Escherichia coli***	***S. aureus***	
Hydroxypropyl methylcellulose (HPMC)	Oregano (0.75%)	31.22 ± 0.24	35.46 ± 0.33	[80]
		***Escherichia coli***		
Alginate–apple puree	Oregano (0.1%)	49.8		[81]
	Cinnamon (0.5%)	40.8		
	Lemongrass (0.5%)	19.6		
		***Escherichia coli***	***S. aureus***	
Polyvinyl alcohol nanofibre	Cinnamon nanophytosome (5%)	12 ± 0.82	12 ± 2.12	[82]
		***Escherichia coli***	***S. aureus***	
Carboxymethyl cellulose	Zataria multiflora Boiss (3%)	56.33 ± 1.52	57.66 ± 1.15	[83]

**Table 3:** Moisture content, water vapor permeability (WVP), Tensile strength (MPa) and elongation at break (%) of anti-microbacterial agent reinforced starch biopolymer.

Starch	EOs	Moisture Content (%)	Water Vapor Permeability (g^−1^·s^−1^·Pa^−1^)	Tensile Strength (MPa)	Tensile Modulus (MPa)	Elongation %	References
Corn/Wheat	Lemon	16.2 ± 0.29	3.28 ± 0.06	11.16 ± 1.03	-	26.50 ± 0.39	[56]
Corn/Wheat	Lemon	11.0 ± 0.33	3.07 ± 0.08	11.50 ± 1.06	-	32.20 ± 0.55	[56]
Cassava	Cinnamon	-	9.78 ± 1.46	1.05 ± 0.16	-	191.27 ± 22.62	[7]
Cassava	Cinnamon (1.5%)	-	14.79 ± 2.76	0.63 ± 0.09	-	108.85 ± 7.29	[57]
Potato	Lavender (2%)	72.28 ± 9.75	-	62.8 ± 1.9	-	19.5 ± 2.6	[58]
Pea	Oregano	4.33 ± 0.13	4.9 ± 0.5	26 ± 2.0	502 ± 50	42 ± 11	[73]
Pea	Neem	6.7 ± 0.13	4.1 ± 0.4	21.5 ± 1.0	413 ± 32	44 ± 7	[73]
Cassava	Lemon Grass (0.5%)	-	23.28 ± 1.17	0.52 ± 0.022	-	30.02 ± 1.46	[1]
Cassava	Tea tree (1.5%)		9.958 ± 1.20	3.03 ± 0.46		25.1 ± 2.8	[61]
Tapioca	Cinnamon bark (2.0%)		21.38 ± 1.29	1.23 ± 0.09		58.56 ± 1.23	[62]
Corn/octenylsuccinated	Soybean (0.5%)		2.72 ± 0.13	5.21 ± 0.20		36.54 ± 6.36	[94]
Potato	Lavandula angustifolia (0.5%)		2.65 ± 0.07	45.88 ± 1.19		15.33 ± 0.15	[74]
Potato	Mentha pulegium (0.5%)		2.59 ± 0.43	36.15 ± 1.91		17.63 ± 2.42	[74]
Soybean	Zataria multi- flora Boiss (ZEO) (3%)	18.92 ± 0.53	1.2 ± 0.05	8.43 ± 0.43		40.66 ± 0.74	[95]
Soybean	Mentha pulegium (MEO) (3%)	27.02 ± 2.07	1.7 ± 0.03	6.72 ± 0.56		38.73 ± 0.36	[95]
Soybean	nano titanium dioxide (TiO_2_-N) (3%)		5.27 ± 0.17	17.22 ± 0.42	0.332 ± 0.032	22.2 ± 4.3	[96]
Chitosan	Mint oil (0.5%)	10.98 ± 0.18	0.028 ± 0.004	16.99 ± 0.46	1.14 ± 0.60	14.87 ± 0.34	[97]
Chitosan	Rosemary oil (0.5%)	5.08 ± 0.50	0.014 ± 0.001	25.95 ± 0.48	1.42 ± 0.53	18.20 ± 0.30	[97]
Fish gelatin/Chitosan nanoparticle	Origanum vulgare L. (1.2%)		0.683 ± 0.050	3.28 ± 0.43	153.75 ± 25.24	87.20 ± 17.14	[78]
Chitosan	Ziziphora clinopodioides (1%)		24.31 ± 0.01	39.31 ± 0.43			[79]
	grape seed (1%)		21.06 ± 0.27	34.48 ± 0.06			
Gelatin	Ziziphora clinopodioides (1%)		24.12 ± 0.18	30.80 ± 0.03			[79]
	grape seed (1%)		20.54 ± 0.24	27.12 ± 0.08			
Hydroxypropyl methylcellulose (HPMC)	Oregano (0.75%)	10.83 ± 0.55	2.53 ± 0.07	25.46 ± 0.06	40 ± 0.34	8.65 ± 0.34	[80]
Alginate–apple puree	Oregano (0.1%)		5.25 ± 0.33	2.47 ± 0.37	5.75 ± 0.96	56.96 ± 3.86	[81]
	Cinnamon (0.5%)		4.90 ± 0.27	2.84 ± 0.48	6.86 ± 1.16	57.88 ± 5.37	
	Lemongrass (0.5%)		4.91 ± 0.40	2.56 ± 0.46	6.02 ± 1.07	55.95 ± 5.55	
Polyvinyl alcohol nanofibre	Cinnamon nanophytosome (5%)	29 ± 2.01	1.07 ± 0.04	45.32 ± 5.06	36.79 ± 4.8	11.07 ± 5.1	[82]
Carboxymethyl cellulose	Zataria multiflora Boiss (3%)	15.64 ± 0.84	5.79 ± 0.29	16.96± 0.95		20.77 ± 0.51	[83]

**Table 4:** Antimicrobial properties of polymer composites reinforced essential oil.

Essential Oil	Properties	Food Product	Film Material	References
Rosemary	Antimicrobial	Chicken	Cellulose acetate	[111]
Cinnamon clove	Antimicrobial	Bakery	Cassava starch	[7]
Lemon, thyme and cinnamon	Antibacterial	NA	Chitosan	[112]
Cinnamon, winter savory and oregano	Antimicrobial	Bologna and ham	Alginate	[113]
Bergamot	Antifungal and antibacterial	NA	Chitosan	[114]
Garlic, rosemary, oregano	Antimicrobial	NA	Whey protein isolate (WPI)	[115]
Oregano	Antimicrobial (against *S. aureus*, *Shewanella putrefaciens* and *Yersinia enterocolitica*) and antioxidants	NA	Quince seed mucilage	[116]
Oregano	Antimicrobial	Pizza	Cellulosic resin	[117]
Oregano	Antimicrobial	Ripened sheep cheese model	Polypropylene (PP) and polyethylene terephthalate (PET)	[118]
Oregano and thyme	Antimicrobial (against *Pseudomonas* spp. and *coliform bacteria*)	Fresh ground beef	Soy protein	[119]
Oregano	Antimicrobial	Fresh beef	Whey protein isolate (WPI)	[120]
Mixture of oregano, pimento berry, and lemongrass (mixture A) Mixture of nutmeg, lemongrass and citral (mixture B)	Antimicrobial (against *L. monocytogenes*)	Fresh broccoli	Methylcellulose (MC) and blend of polycaprolactone/alginate (PCL/ALG)	[121]
Ginger, turmeric and plai	Antioxidant	NA	Fish skin gelatin	[122]
*Satureja hortensis*	Antibacterial and antioxidant	NA	k-Carrageenan	[123]
Oregano and pimento	Antioxidant	Whole beef muscle	Milk protein	[124]
Clove	Antibacterial	Fish	Gelatin chitosan	[125]
Clove (57)	Antibacterial and antioxidant	Sardine patties	Sunflower protein concentrate	[126]
Linalool or methyl chavicol (39)	Antimicrobial	Cheddar cheese	Low-density polyethylene (LDPE)	[126]
Oregano and bergamot (58)	Antimicrobial	Formosa plum	Hydroxypropyl methylcellulose and limonene constituent EO Tomatoes	[127]
Cinnamon and mustard	Antibacterial	Tomatoes	Zein	[128]
Limonene constituent EO, lemongrass and oregano	Antimicrobial	Strawberries	Chitosan	[129]
Peppermint, red thyme, chitosan and lemon	Flavoring	Strawberry	Chitosan	[130]

NA = not available.
